# Derivation and characterization of retinal pigment epithelium from urine‐derived iPSCs

**DOI:** 10.1002/2211-5463.70246

**Published:** 2026-04-24

**Authors:** Daniella Beiner, Hainan Zhu, Carol Christine Bosholm, Heuy‐Ching Wang, Tracy Criswell, Anthony Atala, Jian‐Xing Ma, Yuanyuan Zhang

**Affiliations:** ^1^ Wake Forest Institute for Regeneration Medicine Wake Forest School of Medicine Winston‐Salem NC USA; ^2^ Combat Casualty Care & Operational Medicine Naval Medical Research Unit–San Antonio (NAMRU‐SA) TX USA; ^3^ Department of Biochemistry Wake Forest School of Medicine Winston‐Salem NC USA

**Keywords:** age‐related macular degeneration, cell therapy, induced pluripotent stem cells, regenerative medicine, retinal pigment epithelium, urine‐derived iPSC, urine‐derived stem cells

## Abstract

Age‐related macular degeneration (AMD), particularly its dry form, is a leading cause of irreversible vision loss due to retinal pigment epithelium (RPE) dysfunction and loss. Addressing this unmet therapeutic need requires non‐invasive strategies for generating patient‐specific RPE cells. This study reports the successful generation and initial characterization of RPE cells derived from urine‐derived induced pluripotent stem cells (u‐iPSC‐RPE). Urine‐derived stem cells (USCs) were isolated from healthy individuals and comprehensively characterized, confirming strong expression of renal progenitor makers and mesenchymal stem cell markers, while lacking standard hematopoietic markers. USCs were reprogrammed into iPSCs using the integration‐free Sendai virus expressing the Yamanaka factors. The reprogrammed u‐iPSC clones displayed characteristic pluripotency marker expression and demonstrated clearance of the Sendai virus by later passages. Subsequently, these u‐iPSCs were efficiently differentiated into RPE cells, exhibiting characteristic hexagonal morphology and pigmentation which was confirmed by the expression of key RPE‐specific proteins. Our findings demonstrate the feasibility and reliability of generating patient‐specific u‐iPSC‐RPE cells from readily accessible USCs providing a foundation for future studies to investigate their functional potential for retinal disease modeling and therapeutic applications for AMD.

## Graphical abstract

Age‐related macular degeneration causes vision loss via RPE dysfunction and loss. Traditional iPSC therapies rely on invasive biopsies, limiting scalability. Here, we utilize urine‐derived stem cells as an accessible source to generate u‐iPSCs, successfully differentiated into pigmented RPE. This “Urine‐to‐Retina” platform provides a promising path for autologous cell therapy and drug screening. *Graphic created using SciDraw AI* (*free license*).

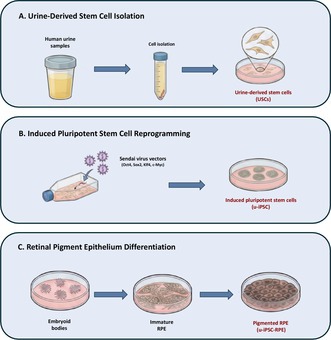

**Abbreviations**
AMD: Age‐Related Macular Degeneration
DIM: Differentiation Induction Media
DPM: Differentiation Propagation Media
EB: Embryoid Body
iPSC: Induced Pluripotent Stem Cells
PEC: Parietal Epithelial Cells
RPE: Retinal Pigment Epithelium
RPEMM: Retinal Pigment Epithelium Maturation Media
SeV: Sendai Virus
USC: Urine‐Derived Stem Cells

Age‐related macular degeneration (AMD) is a leading cause of irreversible vision loss in the United States [1, 2, 3] and affects close to 200 million individuals worldwide [3, 4]. Dry AMD accounts for approximately 80% of all AMD cases [5] and can progress to geographic atrophy characterized by retinal pigment epithelium (RPE) cell death, subsequent photoreceptor loss, and ultimately profound vision impairment. Currently, no effective approaches exist to prevent RPE and photoreceptor degeneration, highlighting the urgent need for innovative therapeutic interventions.

The advent of induced pluripotent stem cell (iPSC) technology has revolutionized regenerative medicine. By reprogramming somatic cells into a pluripotent state, iPSCs offer an unprecedented opportunity to generate patient‐specific cell types for therapeutic transplantation, disease modeling, and drug screening. The potential of iPSC‐derived RPE (iPSC‐RPE) transplantation to restore retinal function in AMD patients is substantial, with initial pioneering clinical trials demonstrating their feasibility [6]. Numerous preclinical investigations involving both iPSC suspensions and monolayers continue to explore their therapeutic promise [7, 8].

Despite their immense potential, several significant challenges currently impede the widespread clinical translation of iPSC therapies, and more specifically iPSC‐RPE therapies. These include the risk of immune rejection associated with allogeneic (non‐self) grafts, even within the immune‐privileged ocular environment, and the invasive and often inefficient process of obtaining current autologous somatic cell sources, such as foreskin fibroblasts [7, 9], peripheral blood mononuclear cells [8, 10], bone marrow cells [11, 12], and nasal mucosa [13] and conjunctival tissues [14, 15]. To address these limitations, our research leverages human primary urine‐derived progenitor or stem cells (USCs), a cell source we initially reported in 2008 [16, 17, 18], as a novel and superior alternative for iPSC generation. USCs, a renal progenitor population, offer an easily accessible and highly regenerative donor cell source [16, 19, 20, 21, 22, 23, 24, 25, 26, 27, 28, 29, 30, 31, 32, 33, 34, 35, 36, 37, 38, 39, 40, 41, 42, 43, 44, 45, 46, 47, 48, 49, 50]. The ability to obtain these cells through a simple urine collection process inherently eliminates the need for invasive procedures, significantly reducing patients' burden and simplifying the logistics of cell procurement. The feasibility of urine‐derived iPSC generation has been previously established [51, 52, 53, 54]; here, we emphasize the use of autologous cells from donors across a wide age range, specifically older donors, and methodology that will enable streamlining of future adaptation for clinical use in AMD treatment. This practical and economical approach to generate autologous iPSC‐RPE intrinsically eliminates the risk of immune rejection, paving the way for a personalized therapy meticulously tailored to each patient's specific needs and disease stage.

While iPSC‐RPE cells have been successfully generated from various somatic sources such as skin [7] and blood cells [8], methods for non‐invasively derived iPSC‐RPE are still being refined. There are clinical reliable protocols that leverage highly accessible cell sources and demonstrate efficient differentiation kinetics to enhance the clinical viability and broad accessibility of this therapy. Our previous work suggests that u‐iPSCs more efficiently differentiate into neural progenitor cells [52], which are the precursor cell population for RPE cells.

In this study, we explored the feasibility of creating RPE cells from iPSCs derived from human urine samples (u‐iPSC‐RPE cells) and aimed to establish a reproducible protocol for generating iPSCs from USCs, rather than somatic renal tubule cells, and subsequently differentiate them into RPE‐like cells. We hypothesized that the differentiated u‐iPSC‐RPE cells would exhibit key morphological features and express crucial protein markers comparable to native RPE cells. To achieve this, we characterized morphology and measured the expression levels of relevant pluripotency markers in u‐iPSCs, as well as RPE‐specific markers in the differentiated u‐iPSC‐RPE cells. This work represents a crucial step toward developing an efficient and reproducible approach for autologous RPE replacement using iPSCs derived from patients' own USCs.

## Materials and methods

### Urine sample collection and USC isolation

Urine samples were collected with the approval of the Wake Forest School of Medicine Institutional Review Board (IRB00014033, 9/21/2010), conforming to the standards set by the Declaration of Helsinki. An informed consent form was distributed and signed by all participants. Urine collection followed a previously established protocol [16]. Briefly, 50–300 mL of fresh and clean‐catch urine samples were obtained from consenting adult healthy male donors (*n* = 8, aged 22–64 years). The samples were centrifuged, and the resulting cell pellet was cultured in individual wells of a 24‐well tissue culture plate. Culture media and supplements were used as previously reported [16], utilizing a mixed medium of keratinocyte‐serum‐free medium and progenitor cell medium in a 1:1 ratio. Briefly, keratinocyte serum‐free medium (Cat. #17005042; Thermo Fisher Scientific., Waltham, MA) was supplemented with 5 ng/mL epidermal growth factor, 50 ng/mL bovine pituitary extract, 30 ng/mL cholera toxin, 100 U/mL penicillin, and 1 mg/mL streptomycin. Progenitor cell medium consisted of 3/4 Dulbecco's modified Eagle's medium and 1/4 Ham's F12 medium, supplemented with 10% fetal bovine serum (FBS, Sigma‐Aldrich‐Aldrich, St. Louis, MO), 0.4 μg/mL hydrocortisone (Sigma‐Aldrich), 10^−10^ m cholera toxin (Sigma‐Aldrich), 5 ng/mL insulin (Sigma‐Aldrich), 1.8 × 10^−4^ m adenine (Sigma‐Aldrich), 5 μg/mL transferrin (Sigma‐Aldrich), 2 × 10–9 m 3,3′,5‐triiodo‐L‐thyronine (Sigma‐Aldrich), 10 ng/mL epidermal growth factor, and 1% penicillin–streptomycin. Unless otherwise specified, all reagents were acquired from Thermo Fisher Scientific.

### Flow cytometry analysis for MSC and HSC markers

For flow cytometric analysis, USCs at passage 4 (p4) were harvested by trypsinization, washed twice with ice‐cold PBS, and resuspended at a concentration of 1 × 10^6^ cells/mL Approximately 1 × 10^5^ cells were then incubated in the dark for 30 min at 4 °C with fluorochrome‐conjugated antibodies targeting a panel of positive and negative mesenchymal stem cell (MSC) and hematopoietic stem cell (HSC) markers. The following antibodies were used: renal progenitor cell markers CD24 and CD133 [55, 56, 57, 58]; MSC markers CD73‐APC, CD90‐PE, CD105‐FITC; and HSC markers CD34‐PerCP‐Cy5.5, CD45‐FITC, VEGFR2. Corresponding isotype‐matched negative control antibodies (e.g., IgG1‐APC, IgG1‐PE, IgG1‐FITC, IgG1‐PerCP‐Cy5.5) were used to define background fluorescence.

Following incubation, USCs were washed twice with ice‐cold PBS containing 0.5% FBS and then resuspended in 300 μL of the same buffer for immediate analysis. Data acquisition was performed on a BD FACSCalibur™ flow cytometer using cellquest™ software. A minimum of 10 000 events were acquired for each sample. Data analysis was performed using flowjo™ software (Tree Star, Inc.). Cells were initially gated based on forward scatter (FSC) and side scatter (SSC) to exclude debris and doublets. Positive expression was determined by setting gates based on the fluorescence intensity of the isotype controls, with expression levels reported as the percentage of positive cells. All reagents were acquired from BD Biosciences (San Diego, CA).

### Generation of urine‐derived iPSCs (u‐iPSCs)

To generate iPSCs, USCs (passage 3–5) were reprogrammed using the cytotune™‐iPS 2.0 Sendai Reprogramming Kit (Cat. #A16517; Thermo Fisher Scientific), which utilizes Sendai virus (SeV) vectors expressing the four Yamanaka factors: OCT4, SOX2, KLF4, and c‐MYC (OSKM). The methodology was based on our previously described protocol [52]. Briefly, USCs were seeded at a density of approximately 2 × 104 cells/well into two wells of 12‐well culture plate two days prior to transduction. On the day of transduction (Day 0), a live cell count was performed on one well using a countess™ Automated Cell Counter, and the appropriate volume of each virus added to the second well to reach the target MOI (KOS MOI = 5, hc‐Myc MOI = 5, hKlf4 MOI = 3). The Sendai viral vector‐containing medium was replaced with fresh medium 24 h post‐transduction.

iPSC colonies typically began to form by Day 5 post‐transduction. Reprogramming efficiency was calculated at Day 7 by number of colonies per cell number prior to induction × 100%, before replating the cells onto vitronectin‐coated culture dishes at a density of 1–2 × 105 cells/cm2 (Cat. # A14700; Thermo Fisher Scientific). The culture medium was then switched to Essential 8™ medium (Cat. #A1517001; Thermo Fisher Scientific) or mTeSR™ Plus medium (Cat. #100‐0276; Stemcell Technologies) for feeder‐free maintenance. Colonies were manually picked and replated on Day 16–21. iPSC were routinely passaged when the colonies covered ~85% of the culture vessel surface area using 0.5 mm EDTA (Cat. #15575020; Invitrogen, Thermo Fisher Scientific) in Calcium and Magnesium‐free DPBS, Gentle Cell Dissociation Reagent (Catalog #100‐0485, Stemcell Technologies) or 0.02% Versene EDTA (Versene Solution, Cat #15040066, Gibco, Thermo Fisher Scientific). For convenience, iPSCs derived from USCs are hereafter referred to as u‐iPSCs.

### Immunofluorescent staining for Sendai virus components and pluripotency markers

To evaluate the clearance of Sendai viral components, iPSC colonies were analyzed by immunofluorescent staining at early passages and compared to later passages (p6). Cells were seeded onto chamber slides (Nunc™ Lab‐Tek™ Chamber Slide System) and allowed to adhere. For staining, cells were fixed with 4% paraformaldehyde (PFA) for 15 min at room temperature, washed twice with DPBS and permeabilized with 0.1% Triton X‐100 in DPBS for 10 min. Non‐specific binding was blocked using 5% normal goat serum (NGS) and 1% BSA in DPBS for 1 h at room temperature. Cells were incubated overnight at 4 °C with primary antibody (Sendai virus HN Monoclonal Antibody 1A6, eBioscience™, Thermo Fisher Scientific) diluted in blocking solution, washed 3× with DPBS and incubated with secondary antibody (Goat anti‐Rabbit IgG (H + L) Highly Cross‐Adsorbed Secondary Antibody, Alexa Fluor™ 488, Thermo Fisher Scientific) diluted in DPBS for 1 h at room temperature. Cells were washed with DPBS 3× prior to imaging on Olympus IX‐83 microscope.

### Characterization of u‐iPSCs pluripotency

The following pluripotency markers were used: mouse anti‐OCT4, anti‐Sox2 (Santa Cruz Biotechnology), anti‐SSEA4, and anti‐Nanog (BD Biosciences). TRA‐1‐60 was used as an additional marker by live and immunofluorescent staining for early screening of iPSC clones. Following primary antibody incubation, cells were washed three times with PBS and incubated with appropriate Alexa Fluor‐conjugated secondary antibodies (Thermo Fisher Scientific) diluted in blocking solution for 1 h at room temperature in the dark. Nuclei were counterstained with DAPI (Cat. #D3571; Life Technologies, Thermo Fisher Scientific) for 5 min. All steps involving fluorescent antibodies were performed with minimal light exposure, with care taken to prevent sample desiccation.

### Karyotype analysis

The chromosomal integrity of the u‐iPSC line was confirmed by karyotype analysis before initiation of RPE differentiation (at passage 18). Chromosome analysis was performed on cultured cells by standard techniques, using G‐banding by trypsin‐Giemsa (GTG) stain [59]. Slide scanning, image capture, and karyotyping of 20 metaphase cells were performed manually using the genasis hiband v8.4 system (Applied Spectral Imaging, Carlsbad, CA).

### Trilineage differentiation of u‐iPSC

u‐iPSC were differentiated post‐confirmation of Sendai virus clearance to the three germ layers (ectoderm, mesoderm, and endoderm) using a STEMdiff™ Trilineage Differentiation Kit (Cat. #05230; Stemcell Technologies) following the recommended product protocol. Following differentiation, cells were fixed for 20 min in 4% PFA, permeabilized for 15 min using 0.2% Triton X‐100 diluted in PBS (Cat. #T9284; Sigma‐Aldrich) and blocked for 30 min using 3% BSA (Blocker BSA 10% in PBS, Cat. #37525; Thermo Scientific) or protein block (Cat. #X090930‐2; Agilent, Serum‐Free Protein Block) at room temperature. Cells were incubated with the following primary antibodies diluted 1:20 (Cat. #S302283‐2; Agilent, Antibody Diluent) overnight at 4 °C: anti‐SOX1 (Cat. #AF3369‐SP; R7D Systems, Human/Mouse/Rat SOX1 Antibody), anti‐Desmin (Cat. #AF3844‐SP; R&D Systems, Human/Mouse Desmin Antibody) and anti‐SOX17 (Cat. #AF1924‐SP; R&D Systems, Human SOX17 Antibody) for differentiated ectoderm, mesoderm and endoderm respectively, followed by incubation with a secondary antibody diluted 1 : 1000 (Donkey anti‐Goat IgG (H + L) Cross‐Adsorbed Secondary Antibody, Alexa Fluor™ 568, Cat. #A‐11057; Thermo Fisher Scientific) for 1 h at room temperature and counterstaining with DAPI (Cat. #D3571; Life Technologies, Thermo Fisher Scientific) for 10 min. Cells were washed for 2 × 5 mins using TBS (Cat. #PPB011; Sigma‐Aldrich, Tris Buffered Saline) and 1 × 5 mins using PBST (PBS with 0.1% Tween20; Cat. #P1379; Sigma‐Aldrich) between each step. All images were taken on Olympus IX‐83 microscope. Triplicate images at 10× magnification were analyzed to assess percentage of positively stained cells by area using imagej.

### Differentiation of u‐iPSCs into RPE cells (u‐iPSC‐RPE cells)

u‐ iPSCs (passage 15–20) were maintained under standard feeder‐free conditions. A previously published protocol was followed to initiate differentiation [60]. In short, iPSCs were digested into small clusters using relesr™ (Cat. #100–0483; Stemcell Technologies). Cell clusters were transferred to Corning Ultra‐Low Attachment 6‐well plates and suspended as embryoid bodies (EBs) in mTeSR Plus (Cat. #100‐0276; Stemcell Technologies) with added 10 μm ROCK inhibitor. Media was replaced after 24 h to remove the ROCK inhibitor and gradually replaced with differentiation induction media (DIM). After 6 days, EBs were replaced on Matrigel‐coated 6‐well plates, and the media was gradually shifted to differentiation propagation media (DPM), followed by Retinal Pigment Epithelium Maturation Media (RPEMM) on Day 22. RPEs were passaged after the onset of pigmentation on Day 40 onto fresh Matrigel‐coated plates and maintained in RPEMM up to Day 60–65. Unless specified otherwise, all reagents and supplements were purchased from Thermo Fisher Scientific.

### Characterization of u‐iPSC‐RPE cells

The differentiated RPE cells were characterized using a multi‐pronged approach to assess their morphology and RPE‐specific protein expression. The morphology of the u‐iPSC‐RPE cells was assessed using phase‐contrast microscopy throughout the differentiation process. Immunocytochemistry was employed to confirm the expression and localization of key RPE proteins: Retinoid Isomerohydrolase (RPE65), Zona Occludens‐1 (ZO‐1) for tight junctions, and Melanogenesis Associated Transcription Factor (MiTF). Cells were fixed with 4% paraformaldehyde, permeabilized with 0.1% Triton X‐100, and blocked with 5% normal goat serum. Primary antibodies used included mouse anti‐RPE65 (RPE65 Monoclonal Antibody, Cat. #MA1‐16578, Abcam, Waltham, MA) and mouse anti‐ZO‐1 (ZO‐1 Polyclonal Antibody, Cat. #40‐2200; Invitrogen, Thermo Fisher Scientific) and rabbit anti‐MiTF (MiTF Recombinant Rabbit Monoclonal Antibody, Cat. #MA5‐32554; Thermo Fisher Scientific).

### Statistical analysis

All experiments were performed with at least three independent biological replicates to ensure reproducibility. Quantitative data from immunostaining were analyzed using imagej for marker‐positive cell percentages. These additions provide a robust framework for interpreting the differentiation efficiency and marker expression in u‐iPSC‐derived RPE cells.

## Results

### Isolation and characterization of urine‐derived stem cells

**Fig. 1:**
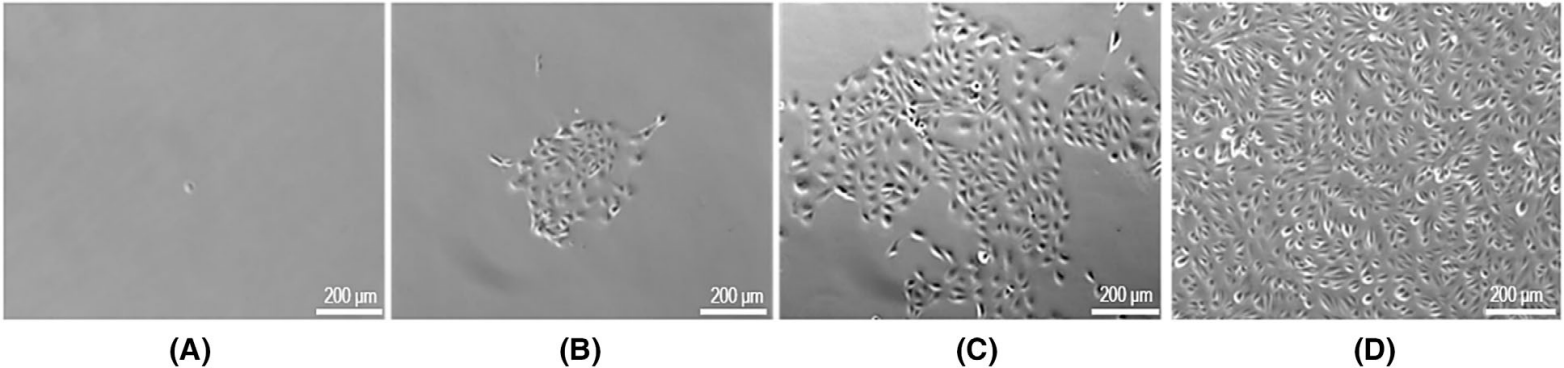
Morphology of Urine‐Derived Stem Cells. (A) Initial stage: single USC at passage 0 (p0) Day 3; (B) Clone formation: USC colony at p0 Day 7; (C) Expansion phase: USCs at p0 Day 10; (D) Subsequent passages: USCs exhibiting characteristic “grain‐of‐rice” appearance. Scale bar: 200 μm.

Urine‐derived stem cells (USCs) were successfully isolated from the collected urine samples of healthy adult donors (*n* = 8, ages 22–64) as described [16]. These isolated cells exhibit characteristics consistent with adult stem cells upon culture. The morphology of USCs underwent distinct changes as they were cultured and expanded *in vitro* (Fig. 1). When initially plated, USCs started as single, isolated adherent cells, appearing by day 3 post‐plating. Within a few days, the single cells have undergone several rounds of division, leading to the formation of clones and the emergence of distinct colonies by day 7. The cells continued to proliferate and displayed the expected spindle‐shaped morphology often described as a “grain‐of‐rice” shape, common to mesenchymal stem cells (MSCs) and other fibroblast‐like cells during proliferation in culture. This progress indicated successful proliferation and establishment of the cell line.

### Phenotypic characterization of USCs by flow cytometry

**Fig. 2:**
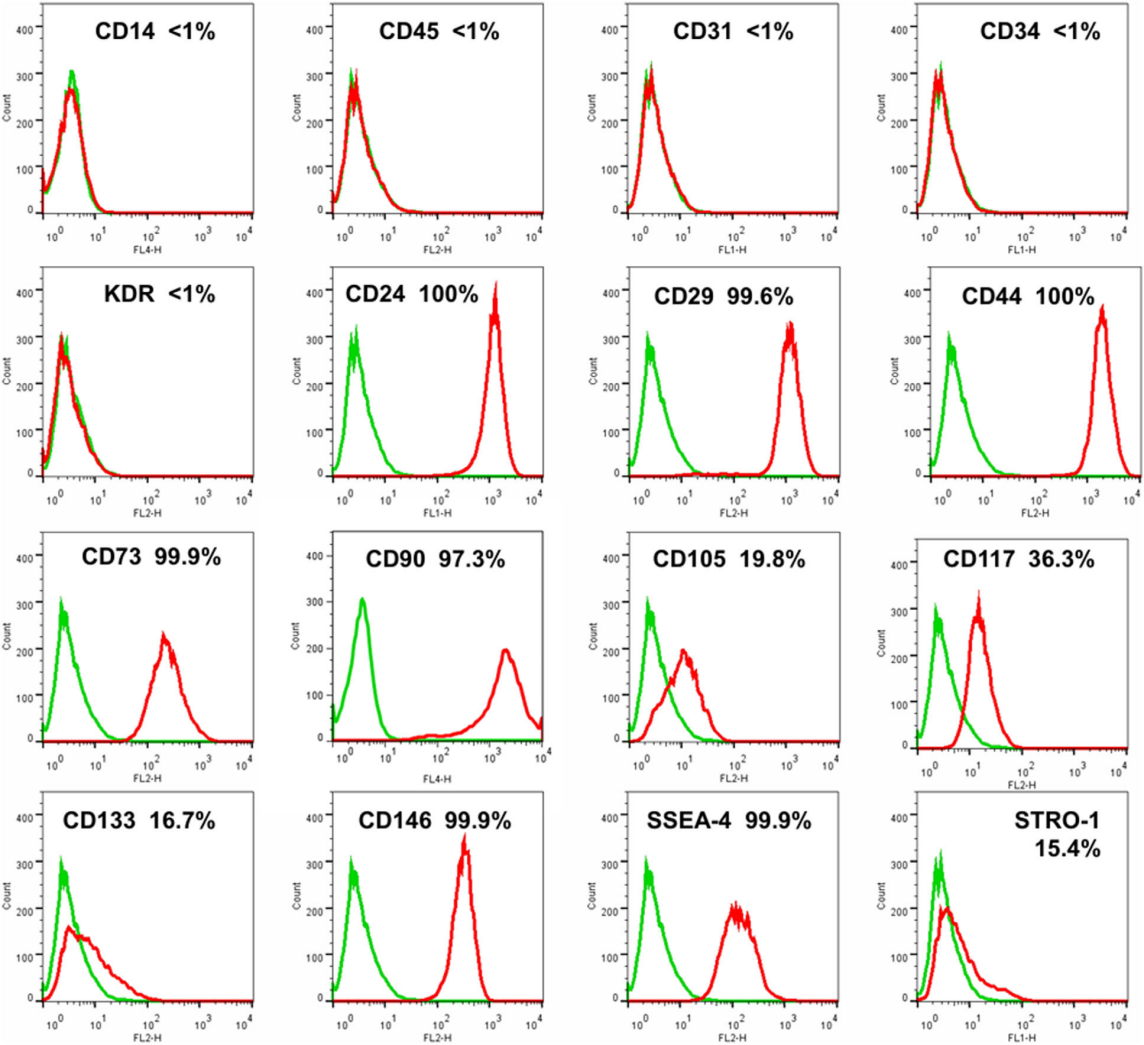
Immunophenotypic Profile of USCs. Flow cytometric analysis of USCs demonstrated robust expression of characteristic renal progenitor markers CD24 and CD133, MSC surface markers, including strong positive for CD29, CD44, CD73, CD90, CD146, and SSEA‐4, moderate positive for CD105, CG117, and Strol‐1. Significantly. These cells were negative for hematopoietic lineage markers CD14, CD31, CD34, CD45 and VEGFR2, confirming their non‐hematopoietic origin and MSC identity.

Flow cytometric analysis was performed on cultured USCs at p4 to characterize their surface marker expression, specifically evaluating the expression of common MSC and hematopoietic stem cell (HSC) markers. Representative flow cytometry plots are shown in Fig. 2. The USCs consistently demonstrated positive expressions of characteristic renal progenitor cell markers, markedly CD24 and CD133 [55, 56, 57, 58], and MSC markers. Specifically, 100% of cells expressed CD24, 16.7% expressed CD133, 99.6% expressed CD29, 100% expressed CD44, 99.9% expressed CD73, 97.3% expressed CD90, and 19.8% expressed CD105 (Fig. 2). These findings are consistent with the International Society for Cell & Gene Therapy (ISCT) minimal criteria for defining multipotent MSCs.

Conversely, the USCs exhibited very low or negligible expressions of hematopoietic and leukocyte surface markers. The percentage of cells positive for CD3 was < 1%, CD34 was < 1%, CD45 was < 1%, CD14 was < 1%, and VEGFR2 was < 1% (Fig. 2). The absence of these markers further supports the non‐hematopoietic origin and mesenchymal nature of the isolated USCs, distinguishing them from hematopoietic stem cells or contaminating immune cells. Collectively, these results confirmed that the urine‐derived cells isolated and expanded under the described conditions possessed a surface immunophenotype highly characteristic of multipotent MSCs.

### Generation and morphology of urine‐derived iPSC

All USC lines were successfully reprogrammed into u‐iPSCs with high reprogramming efficiency ranging between 1.26–1.91% (*n* = 4), demonstrating the robust reproducibility and efficiency of our protocol. Notably, there was no significant difference observed between the reprogramming efficiency of cell lines based on donor age. Average reprogramming efficiency of older donor cells (*n* = 2, age > 50 years, 1.59% reprogramming) was comparable to that of younger donor cells (*n* = 2, age < 50 years, 1.58% reprogramming). These findings establishing the suitability of this method for autologous u‐iPSC generation across a wide age range are particularly relevant given that age is an important consideration for future translational applications in AMD treatment. Future studies with larger cohorts and quantitative analyses will help further characterize these effects and support optimization of the protocol for clinical translation.

**Fig. 3:**
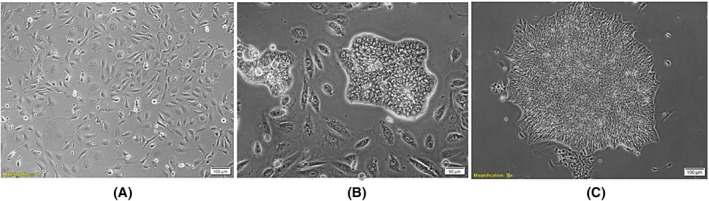
Morphology of Urine‐Derived iPSC Colonies. (A) Urine‐derived stem cells (USCs) at passage 3 (p3) before Sendai virus (SeV) transfection, serving as a control. Scale bar: 100 μm. (B) Early u‐iPSC aggregates on Day 5 after SeV transfection. Scale bar: 500 μm. (C) u‐iPSC colony at passage 9 (p9) Day 3. Scale bar: 100 μm.

Reprogrammed u‐iPSC colonies became visible after approximately five days post‐transduction and displayed the characteristic iPSC morphology. These aggregates were presented as relatively round colonies with sharp, well‐defined edges (Fig. 3B). High‐magnification imaging revealed tightly packed cells exhibiting a high nucleus‐to‐cytoplasm ratio and prominent nucleoli. Colony centers often appeared as dense areas with increased brightness under phase‐contrast microscopy (Fig. 3C).

### Monitoring the clearance of Sendai viral components in reprogrammed u‐iPSCs

**Fig. 4:**
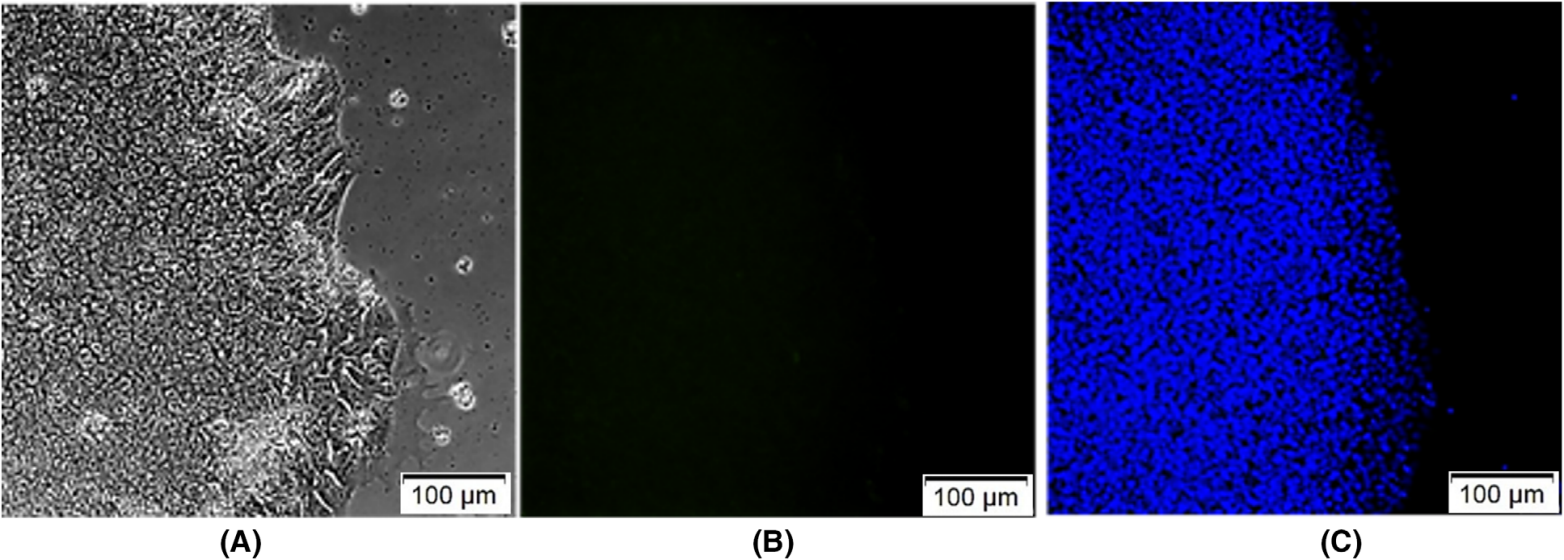
Loss of Sendai Viral Protein Expression in u‐iPSC Lines. (A) Phase contrast of u‐iPSC at p6; (B) Immunostaining with anti‐SeV antibody showing absence of viral protein expression; (C) Merged image of anti‐SeV antibody staining with DAPI counterstain. All images taken at magnification 5×. Scale bar: 100 μm.

A critical step for generating clinically relevant iPSCs is ensuring the complete clearance of any reprogramming vectors. This makes the use of Sendai virus for iPSC generation advantageous due to its non‐integrating nature, significantly enhancing the resulting iPSCs' safety for potential therapeutic applications. We monitored the clearance of Sendai viral components via immunofluorescence staining at various passages. Our analysis employed an anti‐Sendai virus antibody, targeting hemagglutinin‐neuraminidase (HN), a viral protein co‐expressed with the reprogramming genes. We observed a progressive reduction in HN over time during culture of the cells. By later passages (p6), the u‐iPSC lines demonstrated a complete absence of detectable signal for anti‐Sendai virus (Fig. 4), confirming their “virus‐free” status. This crucial finding indicates that exogenous reprogramming genes were no longer expressed in the final u‐iPSC lines.

**Fig. 5:**
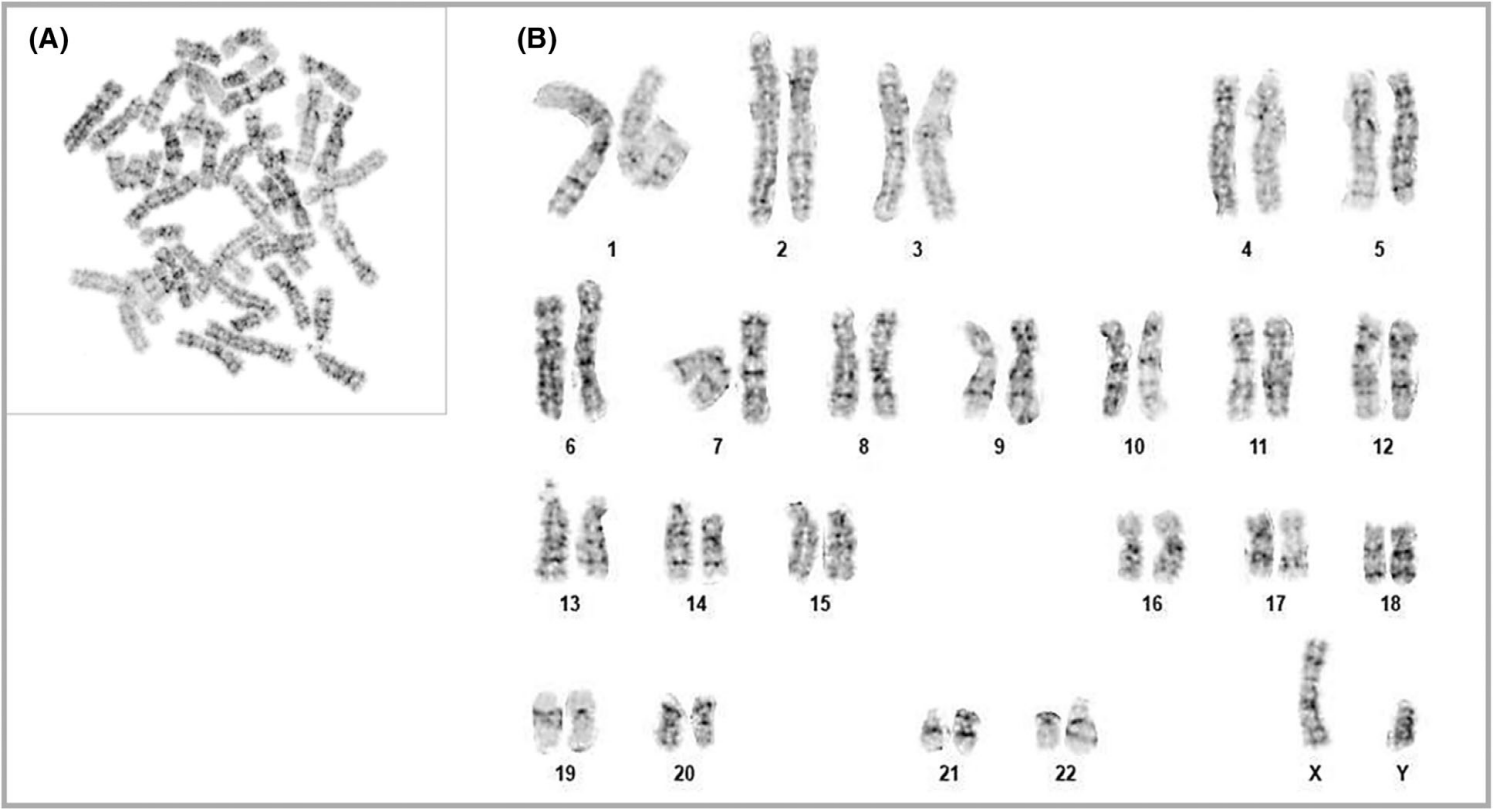
Karyotype of u‐iPSC. (A) Metaphase spread of male urine‐derived iPSC. (B) Karyotyping corresponding to the metaphase spread shown in A. Karyotyping is consistent with a normal male karyotype (46 chromosomes, XY).

To confirm the safety of this reprogramming method, karyotype analysis was utilized to ensure that no chromosomal abnormalities are introduced during the transduction process. The karyotype of male urine‐derived iPSC was consistent with a normal male profile, displaying 46 chromosomes, XY (Fig. 5).

### Characterization of u‐iPSCs

**Fig. 6:**
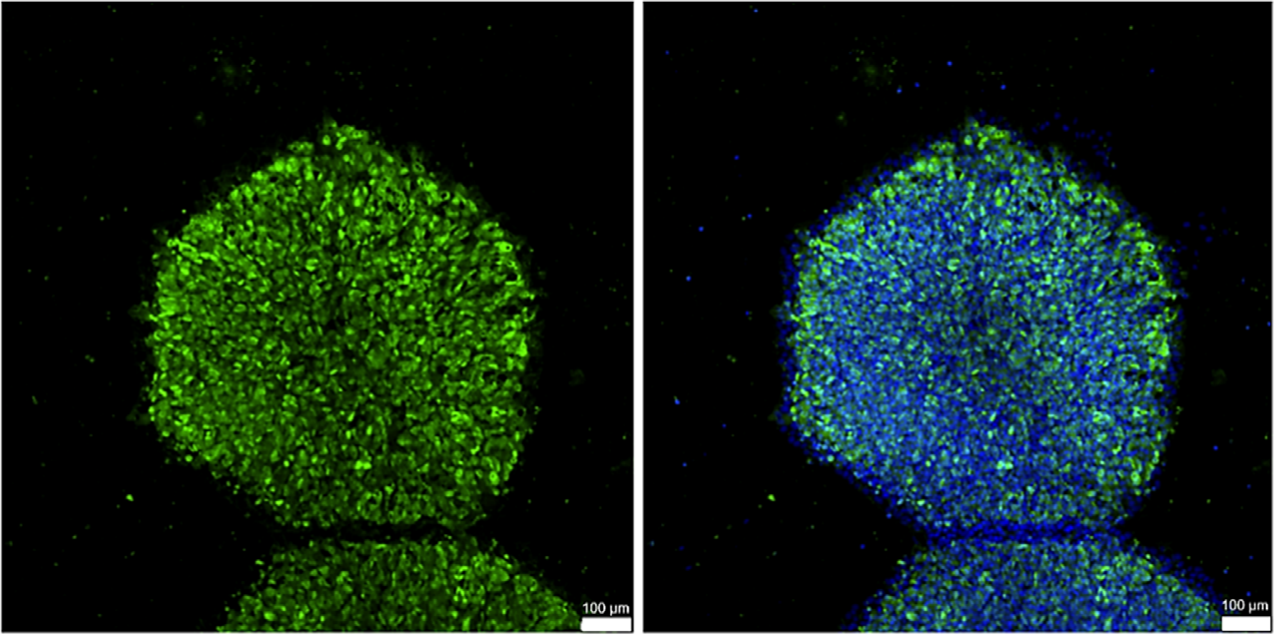
Pluripotent State of Urine‐Derived iPSC. Immunofluorescence staining of u‐iPSC at p12 with key pluripotency marker TRA‐1‐60 (left); double staining for TRA‐1‐60 and DAPI (nuclear stain), positively identifying the iPSC colony (right). Scale bar: 100 μm.

**Fig. 7:**
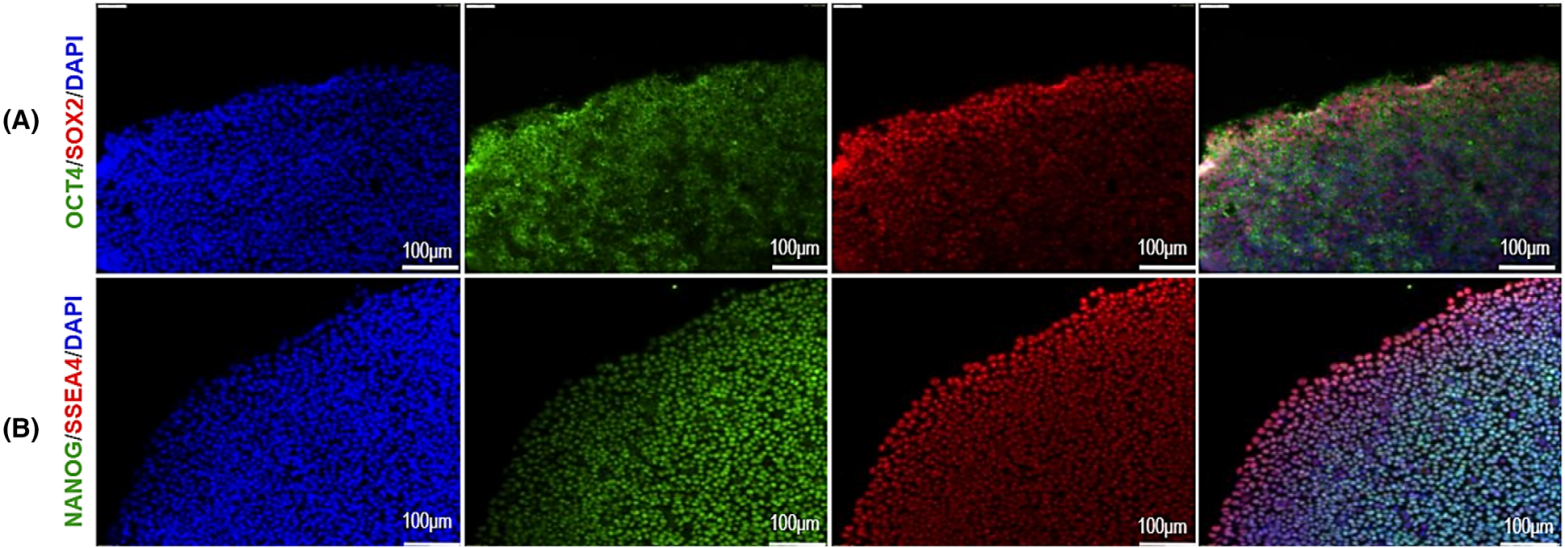
Characterization of Pluripotency Markers in u‐iPSC Lines. Immunofluorescence staining of u‐iPSC displaying prominent nuclear expression of transcription factors (A) SOX2, OCT4, and (B) NANOG, along with strong surface expression of SSEA4. Scale bar: 100 μm.

**Fig. 8:**
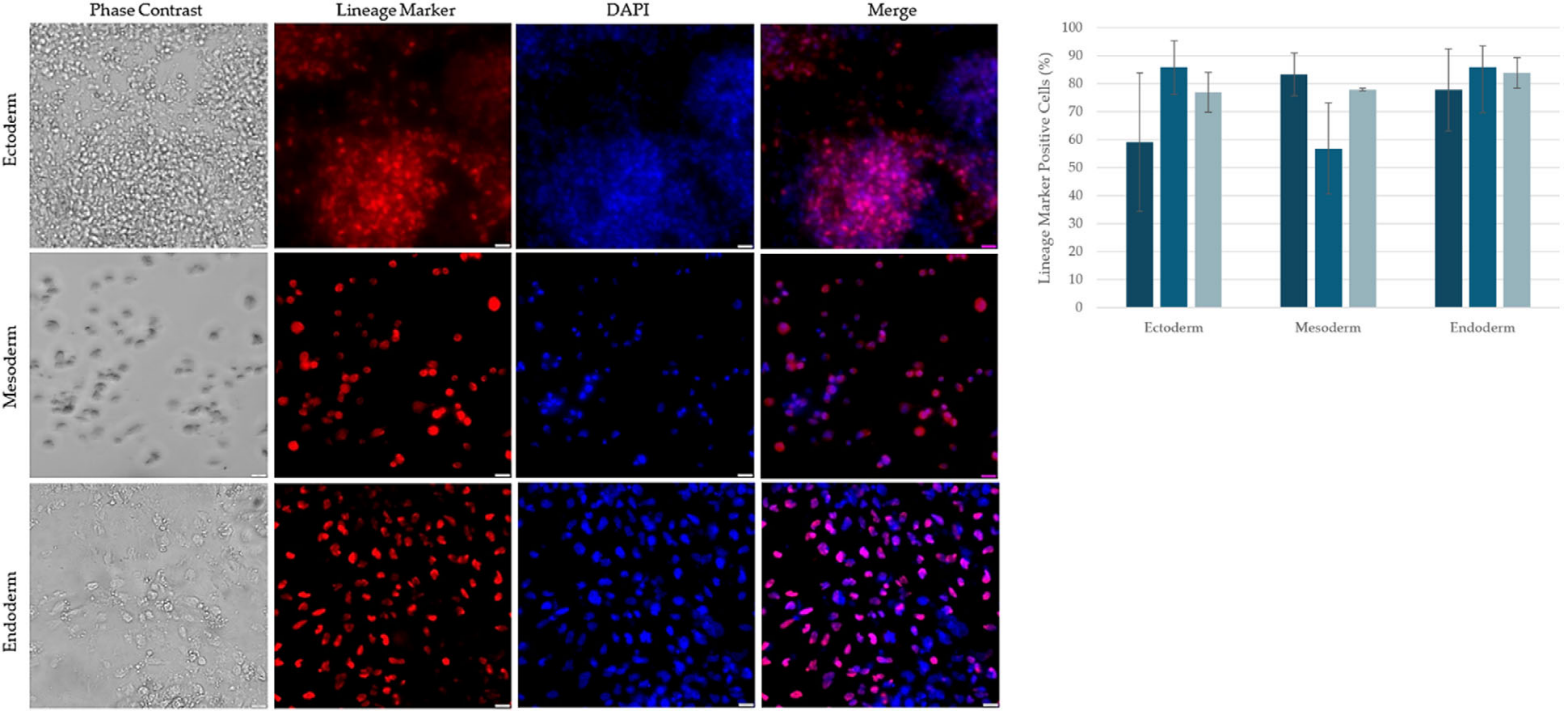
Trilineage Differentiation Capability of u‐iPSC. Pluripotency of u‐iPSC confirmed by differentiation into all three germ layers, assessed by immunofluorescence staining of generated ectoderm, mesoderm, and endoderm for lineage markers SOX1, Desmin, and SOX17 respectively (left). Scale bar: 20 μm. Quantification of positively stained cells for 3 separate cell lines with error bars indicating standard deviation (right).

To confirm their pluripotent state, immunofluorescence staining was performed for key pluripotency markers. u‐iPSCs expressed TRA‐1‐60, a surface marker expressed on pluripotent cells (Fig. 6) and exhibited robust nuclear expression of the transcription factors SOX2, OCT3/4, and NANOG and surface expression of SSEA4 (Fig. 7).

u‐iPSC also demonstrated differentiation capability into all three germ layers, with 28.03%, 80.46%, and 29.83% of differentiated cells staining positive for ectoderm marker SOX1, mesoderm marker Desmin and endoderm marker SOX17 respectively (Fig. 8).

### Differentiation and initial characterization of u‐iPSC‐derived RPE cells

**Fig. 9:**
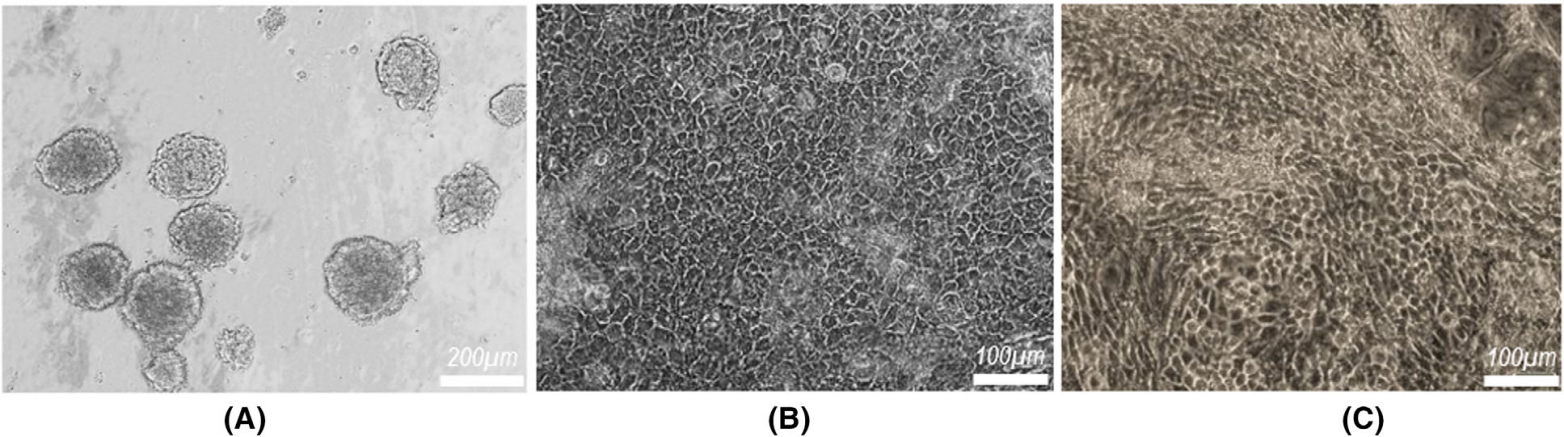
Progressive Morphological Changes of u‐iPSC‐RPE. (A) Embryoid bodies (EBs) 3 days post‐differentiation. Scale bar: 200 μm. (B) RPE with characteristic hexagonal shape in cobblestone‐like arrangements 60 days post‐differentiation. Scale bar: 100 μm. (C) Pigmented RPE with increased cellular density and maturation. Scale bar: 100 μm.

**Fig. 10:**
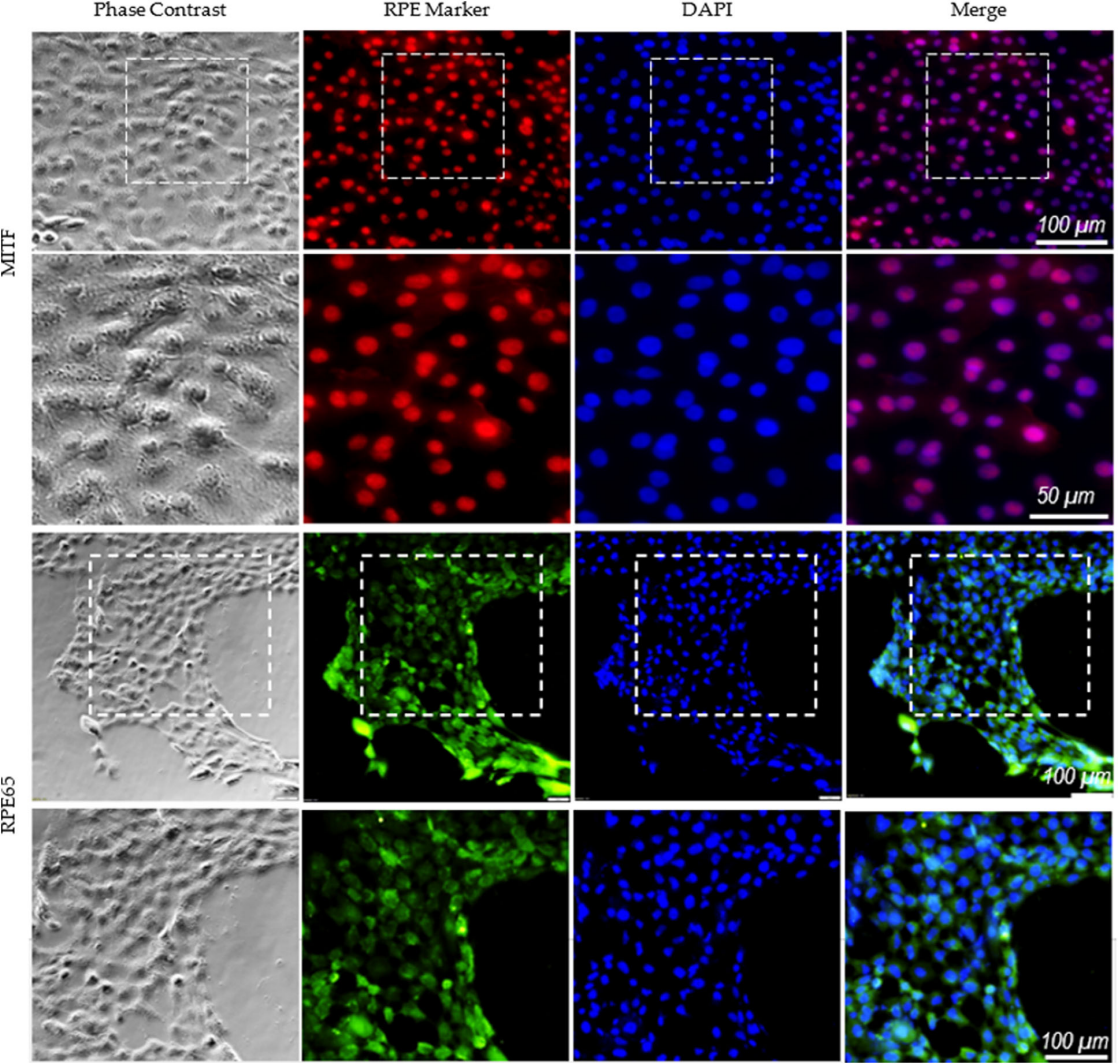
RPE Marker Expression of u‐iPSC‐RPE. Immunofluorescence analysis of u‐iPSC‐RPE post‐differentiation showing protein expression and localization of RPE commitment marker MITF (scale bar: 100 μm, inset panel scale bar: 50 μm) and maturation marker RPE65 (scale bar: 100 μm). Both markers exhibit uniformly high expression in areas of differentiation equivalent to a level of 4+ on a semi‐quantitative scoring scale (1+, < 25%; 2+, 25–50%; 3+, 50–75%; 4+, > 75%).

**Fig. 11:**
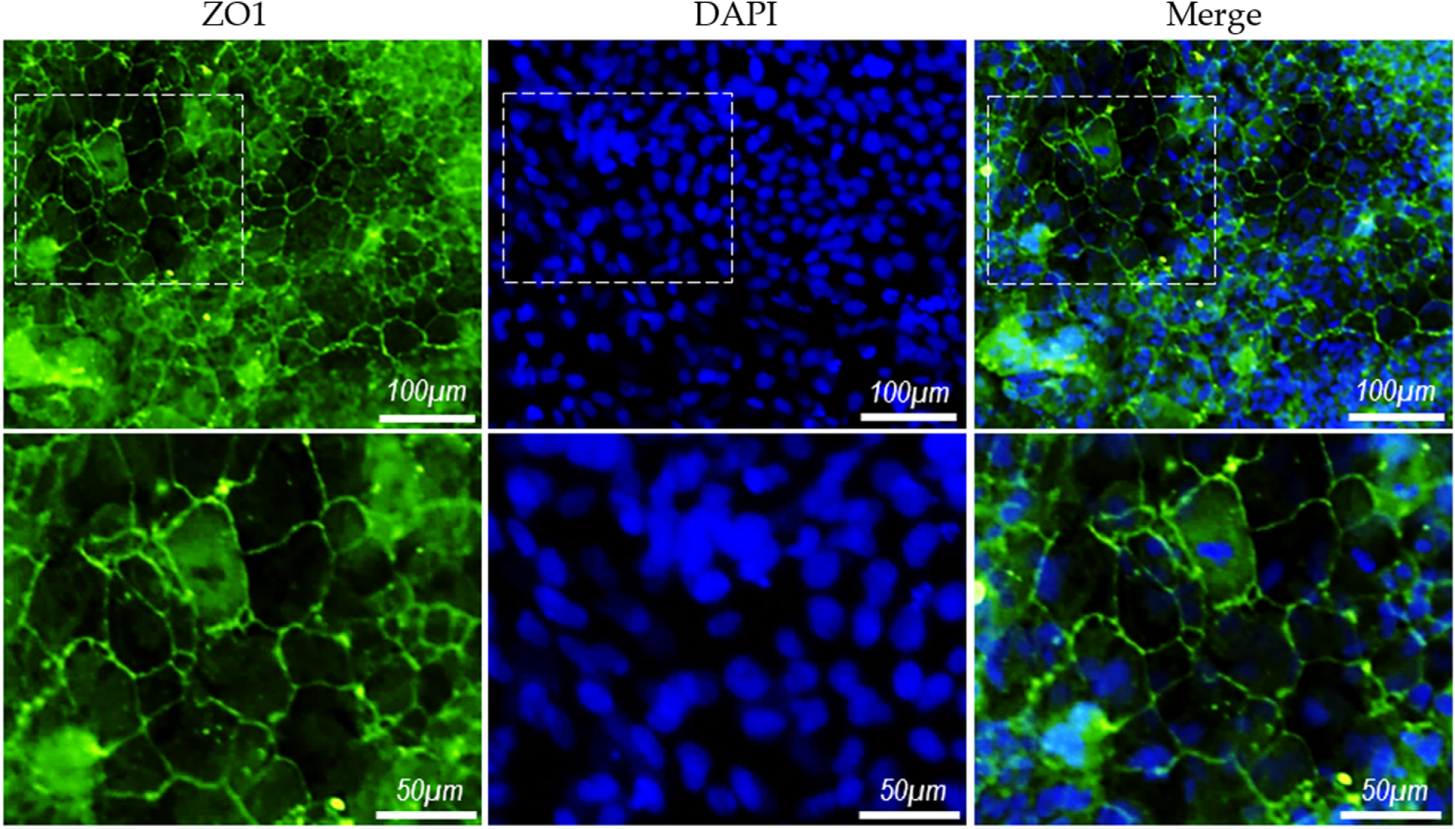
RPE Marker Expression of u‐iPSC‐RPE. Immunofluorescence analysis of u‐iPSC‐RPE post‐differentiation showing protein expression and localization of tight junction marker ZO1. Scale bar: 100 μm, inset panel scale bar: 50 μm.

The established u‐iPSC lines were subjected to a direct differentiation protocol to generate RPE cells. Throughout the differentiation process, we closely monitored the formation of key morphological hallmarks of RPE maturation. In the first 3–5 days after the start of differentiation, the cells began forming embryoid bodies (Fig. 9). Several weeks post‐differentiation, cells exhibited characteristic cobblestone‐like arrangements and showed visible pigment (Fig. 9). Immunocytochemistry was employed to confirm the expression of essential RPE‐specific proteins. Immunofluorescence analysis demonstrated that the differentiated cells robustly expressed and correctly localized the key RPE markers MITF, RPE65 (Fig. 10), and ZO‐1 (Fig. 11).

Collectively, these findings demonstrate the successful differentiation of u‐iPSCs into RPE cells that exhibit characteristic morphology and express a panel of crucial RPE‐specific markers, indicating their potential for further functional maturation. While beyond the scope of this initial characterization, future studies will investigate the functional capabilities of these u‐iPSC‐RPE cells, including RPE65 isomerase activity and their ability to produce 11‐cis retinol.

## Discussion

In this study, we successfully established a reliable and non‐invasive method for generating u‐iPSC‐RPE cells. Our findings demonstrate that stem cells, as opposed to other somatic cells in urine, are responsible for iPSC generation. These u‐iPSCs exhibit characteristic pluripotency and can be efficiently guided toward an RPE lineage, expressing key morphological and molecular markers of RPE.

The choice of the initial cell source for iPSC generation significantly impacts the efficiency, safety, and therapeutic potential of the derived cells. While various somatic cells like skin fibroblasts, PBMCs, bone marrow cells, and nasal or conjunctival tissues have been explored, each presents unique challenges in terms of invasiveness and accessibility. Our work highlights the distinct advantages of using urine‐derived stem cells (USCs) as a source for iPSC generation. Urine collection is non‐invasive, cost‐effective, and provides an easily accessible and virtually unlimited source of cells from healthy adult donors. This streamlined collection process significantly reduces patient burden and enhances the feasibility of generating autologous iPSCs for personalized therapeutic applications in conditions like AMD.

USCs are of epithelial lineage, most likely originating from glomerular parietal epithelial cells (PECs) [17]. PECs, which line Bowman's capsule in the renal glomerulus, exhibit distinct differentiation patterns based on their location. Specifically, PECs located near the vascular pole are thought to act as progenitors for podocytes, while those at the tubular pole are involved in generating renal tubular epithelial cells [55, 61, 62, 63, 64, 65]. Due to their embryonic origin from the intermediate mesoderm [66] and the occurrence of epithelial‐mesenchymal transition (EMT), a process that promotes mesenchymal traits [67], USCs express MSC markers (Fig. 2). They also express strong renal progenitor makers (CD24 and CD133 [55, 56, 57, 58]) and are negative for hematopoietic stem cell markers, results consistent with similar serial studies from our lab [16, 17, 18] and others. Our previous findings also indicate that USCs possess inherent “stemness” properties, including high telomerase activity and relatively long telomeres [68], in addition to the capability of being differentiated down several cell lineages to obtain a variety of cell types, including epithelial cells [37, 69, 70], endothelial cells [71], skeletal myocytes [72], osteocytes [68, 73] and chondrocytes [70]. These characteristics are highly favorable for efficient cellular reprogramming and robust proliferative capacity, offering significant advantages over other somatic cells for efficient iPSC generation.

The precise identification of the starting cell population for urine‐derived iPSCs (u‐iPSCs), and ultimately RPE (u‐iPSC‐RPE), generation is essential. Previous studies on u‐iPSC often lack clarity regarding the specific cell type in urine used for reprogramming, such as exfoliated renal tubule epithelial cells [53, 54] or mesenchymal stromal cells [74]. Urine contains a heterogeneous mix of cells shed from the urinary tract (kidney tubules, bladder, ureters, urethra), with a majority of differentiated epithelial cells and a smaller population of progenitor or stem cells [16]. Although the reprogramming of somatic cells into iPSC is theoretically feasible, the efficiency varies greatly depending on the initial cell type and its original differentiation state. This study, however, clearly demonstrates that the cells utilized for iPSC generation under our culture conditions are of stem cell lineage, rather than somatic cells. Somatic renal tubule epithelial cells are typically lost after subculture [16] and are not sustainable in long‐term culture; only USCs consistently proliferate rapidly through multiple passages in our low‐serum (2.5% FBS) culture system (Fig. 1). Stromal cells are capable of extensive passaging; however, they do not possess the stemness features (i.e., renal progenitor makers, MSC markers, telomerase activity, and multiple differentiation capacity) observed in our cells. This understanding of our starting material helps ensure efficient and consistent reprogramming, resulting in high‐quality iPSCs.

For downstream applications requiring sufficient cell numbers, USCs at passage 3 (p3) were utilized for reprogramming. These cells exhibited robust proliferative capacity, yielding an average of over 1 × 10^7^ cells and approximately 10 clones per 200 mL of urine by p3 within just 3–4 weeks. This impressive expansion highlights their potential as an abundant and accessible cell source for iPSC generation, which can be used to further induce RPE differentiation. Moreover, our study suggests that USCs may exhibit faster reprogramming into iPSCs (6–10 days for iPSC colony formation) compared to other somatic cells, such as skin fibroblasts or PBMCs, which typically require 4 weeks (Table 1) [51, 75]. This comparison underscores the unique benefits of USCs, including their non‐invasive collection, high reprogramming efficiency, and suitability for repeated sampling, which are particularly relevant for patient‐specific and regenerative applications. In contrast, fibroblasts require invasive skin biopsies and extended culture expansion, while PBMCs, although minimally invasive, often exhibit lower and more variable reprogramming efficiencies. This accelerated timeline provides additional downstream benefits to using urine‐derived iPSC for RPE generation.

**Table 1:** Comparative summary of iPSC reprogramming of USCs, fibroblasts, and PBMCs.

Feature	USCs (urine collection)	Fibroblasts (skin biopsy)	PBMCs (blood draw)
Invasiveness	Non‐invasive (Best)	Minimally invasive	Minimally invasive
Repeat access	Easy, no side effects (Best)	Requires repeat biopsy	Requires repeat blood draws
Reprogramming efficiency	Highest (1.2–1.9%)	Lowest (0.02–1.2%)	High (0.8%)
First iPSC colony appearance	Day 5 (Fastest)	Day 12	Day 12
Reprogramming kinetics	Fastest (2 weeks)	Longest (~4 weeks)	Moderate (3–4 weeks)

Abbreviations: iPSCs, Induced Pluripotent Stem Cells; USCs, Urine‐derived Stem Cells; PBMCs: Peripheral Blood Mononuclear Cells.

Our iPSC generation protocol [52], employing Sendai viral vectors for reprogramming, consistently yielded u‐iPSCs that displayed typical iPSC morphology and robust expression of pluripotency markers. A key advantage of this approach is the non‐integrating nature of Sendai virus (SeV). As an RNA virus, SeV completes its life cycle exclusively in the cytoplasm, thus preventing genomic integration and eliminating the risk of insertional mutagenesis. This feature ensures the production of “transgene‐free” iPSCs, where the viral genetic material is naturally cleared during cell division, thus addressing concerns about residual viral DNA and reducing potential long‐term immunogenic risks, all while maintaining high reprogramming efficiency across diverse cell types. An important aspect of our methodology was the rigorous monitoring and confirmation of Sendai viral vector clearance, ensuring the generated u‐iPSCs were “virus‐free” and “transgene‐free” by later passages (Fig. 4). This non‐integrating approach is paramount for the safety and clinical translation of iPSC‐based therapies, addressing concerns associated with genomic integration from other reprogramming methods.

Once reprogrammed, picking u‐iPSC colonies at an early stage is a critical step for successful expansion. In this study, we aimed to identify colonies matching the typical morphology of healthy, undifferentiated iPSC colonies: round, larger and sharp‐edged, with large nuclei. In general, the use of multiple independent markers of iPSCs (such as SOX2/ OCT4/DAPI, and NANOG/SSEA4/DAPI used in this study) for confirmation of pluripotency is considered more precise and provides a higher level of confidence in identifying iPSCs compared to single or double staining.

The subsequent differentiation of these u‐iPSCs into RPE cells resulted in cells exhibiting the characteristic hexagonal morphology and pigmentation of mature RPE (Fig. 9). The expression and correct localization of key RPE‐specific proteins, including RPE65, MITF, and ZO‐1, confirmed by immunocytochemistry (Figs 10 and 11), unequivocally establish the capacity of u‐iPSCs to differentiate into RPE. This aligns with and builds upon previous studies that have successfully differentiated iPSCs from other somatic cell sources into RPE cells [6, 60, 74]. The successful differentiation of u‐iPSCs into RPE cells with these key characteristics of native RPE suggests their potential for therapeutic application in replacing damaged or lost RPE in dry AMD. Quantitative analyses will be performed in our ongoing studies to strengthen reproducibility and enable comparison with established RPE differentiation protocols. Specifically, we plan to perform western blotting, flow cytometry and immunostaining‐based measurements of key RPE markers (e.g., Best1+, MITF+, RPE65^+^, and ZO‐1^+^ cells) to confirm protein‐level expression and assess relative abundance. These quantitative approaches are critical for validating the maturity and functionality of u‐iPSC‐derived RPE cells and will provide a more rigorous framework for benchmarking against existing protocols.

The current study represents an essential early‐stage investigation to establish the u‐iPSC–derived RPE model, with a primary focus on confirming the successful differentiation of u‐iPSCs into RPE cells. This initial milestone is supported by the presence of characteristic RPE morphology and the expression of key RPE‐specific markers. Functional validation will be critical for establishing the translational relevance of this platform, and comprehensive functional studies are already planned as part of our ongoing work. These analyses include (i) assessment of barrier integrity through transepithelial electrical resistance (TEER); (ii) evaluation of essential RPE functions such as phagocytosis of photoreceptor outer segments and visual cycle activity (e.g., 11‐cis‐retinal production); and (iii) quantification of growth factor secretion, including pigment epithelium–derived factor (PEDF) and vascular endothelial growth factor (VEGF). Future studies will further define the functional maturity of u‐iPSC‐RPE cells in both *in vitro* and *in vivo* systems. In addition, to benchmark the functional maturity of u‐iPSC‐RPE against other established iPSC‐RPE platforms, we will perform parallel assessments comparing u‐iPSC‐RPE with fibroblast‐iPSC‐RPE and PBMC‐iPSC‐RPE. Finally, addressing donor‐to‐donor variability and optimizing the scalability of cell production will be important steps toward advancing this model for translational and clinical applications.

For successful clinical translation of u‐iPSC‐derived RPE cells, adherence to regulatory standards is essential. To facilitate future adaptation for clinical use, we have already incorporated xeno‐free culture conditions for u‐iPSC maintenance, including the use of chemically defined media such as Essential 8 and mTeSR Plus, as well as recombinant human Vitronectin as a feeder‐free coating matrix. Sendai virus–based reprogramming was employed to ensure non‐integrating delivery of reprogramming factors, and clearance of exogenous viral genes was confirmed by passage 6 (Fig. 4). Karyotypic stability was assessed, and the findings confirmed that cell lines maintained a normal chromosomal profile. Future translational efforts will focus on transitioning u‐iPSC‐RPE production from research‐grade methods to GMP‐compliant workflows, including donor eligibility screening, exclusive use of GMP‐grade reagents, detailed batch documentation, comprehensive quality control for genetic stability and identity, sterility and endotoxin testing, establishment of master and working cell banks, and implementation of reproducible standard operating procedures. These measures, together with appropriate regulatory oversight, will be critical for meeting safety and quality standards for AMD therapy and enabling clinical trial approval.

## Conclusions

Our findings underscore the use of urine‐derived iPSCs and the promise of autologous u‐iPSC‐RPE cells for future therapeutic applications in AMD. The ease of urine collection, coupled with the robust reprogramming and differentiation efficiency of multipotent USCs demonstrated in this study, positions u‐iPSC‐RPE as a highly attractive and non‐invasive source for patient‐specific cell therapy. Beyond direct clinical applications, u‐iPSC‐RPE also presents a versatile platform for disease modeling and drug screening, offering enhanced fidelity in recapitulating native RPE structure and function. The improved understanding of AMD pathophysiology facilitated by these cells holds promise to accelerate the identification and screening of novel therapeutic agents. Our overarching goal is to develop an efficient u‐iPSC‐RPE‐based therapy for AMD by optimizing donor cell selection and patient targeting, including the investigation of u‐iPSC‐RPE derived from USCs from AMD patients with or without specific gene mutations. Following successful preclinical validation, we envision paving the way for human clinical trials, ultimately revolutionizing AMD treatment and offering hope to millions of patients worldwide through an innovative, non‐invasive, and highly personalized therapeutic option.

## Conflicts of interest

The authors declare no conflicts of interest.

## Author contributions

Conceptualization, YZ; methodology, DB, HZ and CB; software, HZ; validation, YZ, DB, HZ and CB; formal analysis, YZ, DB and HZ; investigation, YZ, DB, HZ and CB; resources, YZ, HCW, JM and AA; data curation, YZ, DB, HZ and CB; writing – original draft preparation, Y.Z.; writing – review and editing, YZ, JM, TC, HCW, DB and AA; visualization, YZ, HCW, DB and HZ; supervision, YZ, JM, AA; project administration, YZ; funding acquisition, YZ. All authors have read and agreed to the published version of the manuscript.

## Data Availability

The data that support the findings of this study is available in Figs 1, 2, 3, 4, 5, 6, 7, 8, 9, 10, 11 and Table 1 of this article.
